# Sustaining women’s empowerment for development in resource-limited settings: an exploratory qualitative approach

**DOI:** 10.3389/frhs.2024.1480784

**Published:** 2024-12-04

**Authors:** Wanno Wallole, Abraham Alano, Misganu Endriyas

**Affiliations:** ^1^ Head Office, South Ethiopia Region Bureau of Labor and Social Affairs, Jinka, Ethiopia; ^2^ School of Public Health, Hawassa University, Hawassa, Ethiopia; ^3^ Health Research and Technology Transfer Directorate, South Ethiopia Region Public Health Institute, Jinka, Ethiopia

**Keywords:** Ethiopia, health development army, sustainability, women’s empowerment, Women’s Development Network

## Abstract

**Background:**

Women's empowerment is one of the fundamental issues for attaining sustainable development goals crossing multiple dimensions. In Ethiopia, Women’s Development Network (WDN), a network of women, was established in 2010 with development aims. Ensuring women’s empowerment critically needs collective efforts of platforms such as WDN. However, there was a paucity of information about the patterns of WDN functionalities, its contribution, and factors affecting its functionality in rural areas of Southern Ethiopia. Hence, this study aimed to explore WDN status and factors affecting their functionality in Southern Ethiopia.

**Methodology:**

The study employed an exploratory qualitative design. Data were collected from purposively selected zones using focus group discussions and key informant interviews. Audio-taped materials were transcribed verbatim and analyzed using a thematic approach. Initially, data were coded (open coding) and after several reviews, themes were developed and interpreted in line with the study objectives.

**Result:**

WDN has passed several steps from its establishment up to now. It was seen skeptically at its early inception, very good level of acceptance in the middle, and staggering currently. However, WDN has contributed to improvements in household welfare resulting from increased ability to afford food, clothing, health, and education. Specific to health, WDN has contributed to general awareness creation, maternal and child health utilization, and environmental sanitation. On the other hand, the volunteer nature of the job put pressure on WDN and revealed socioeconomic stresses. Moreover, inconsistent support from stakeholders especially health extension workers, inadequate men’s engagement, and sidelining of WDN by some educated women remain challenges for the sustainable functionality.

**Conclusion:**

WDN has contributed to multidimensional development goals, especially health services uptake and environmental sanitation*.* However, over time, it became flaccid and lost adequate emphasis from most of its stakeholders and supportive structures. Therefore, considering such vital inputs from community participation in resource-limited settings, stakeholders should offer adequate attention to WDN in terms of selection, training, orientation, follow-up, and acquainting with the community they serve. Moreover, efforts are needed to retain women voluntarily serving and build positive images across all stakeholders and fellow women receiving the services.

## Introduction

Universal development is precisely unattainable without proper women’s involvement in all dimensions of human endeavors. No society can achieve its potential with half its population marginalized and disempowered (1, 2). Global communities have given special emphasis to putting women’s issues at the center of the development as witnessed by the International Conference on Population and Development (ICPD). Women’s involvements have unique capacities in accelerating global development from many perspectives (3).

Women's economic empowerment is the process of achieving equal access to and control over economic resources, ensuring they can use them to exert increased control over other areas of their lives (4). Targeting women as beneficiaries of development programs is only one part of putting them at the center of development. The other part is recognizing their role as agents of change (5). Women tend to invest more of their earnings than men do in their family's well-being as much as 10 times more (6–8). This is a critical fact to consider about the roles of women and girls in development.

However, the status of women and their levels of involvement in most development-oriented issues are inadequate. It is clearly revealed that the level of women’s development-related issues is diverse. Developed nations have relatively better women’s involvement when compared to their developing nation counterparts. In the case of least developed nations, the continuation of the vicious cycle of poverty and women’s alienation from the development arena are some of the causes for the perpetual occurrences of underdevelopment in these regions of the world (9).

The bulk of women’s underdevelopment/disempowerment is observed in the least developed nations of Southeast Asia and sub-Saharan Africa. Ethiopia, being one of the sub-Saharan African nations, bears the burden of women’s underdevelopment or disempowerment (3).

Cognizant of the pervasive burden of women’s underdevelopment and disparity due to the underlying social, economic, cultural, and political situation, Ethiopia has been making significant strides to ameliorate the situation. Several steps have been taken and still proceed. The current constitution has given remarkable consideration to its human rights part with special emphasis on the rights of women. The constitutions articulate the issue under article 35 sub-article 1–3 enshrining that women have equal rights with men in all social, economic, political, and cultural dimensions and they deserve special affirmative actions to balance the underlying disparities (10). Following the constitutional endorsement, several policies and strategies were developed to ease women’s empowerment issues. Women’s affairs policy was developed in 1993 to ensure the capability of women in all development horizons (11). All stakeholders are advised to pay special attention to women's issues in their development-related undertakings.

With the intention to further stretch the organization to the grassroots level and then to improve access to women in most disadvantaged rural areas, the country has designed women’s community-based health extension workers (HEW) to provide preventive health services (12, 13). Its extension further entailed a Women’s Development Network (WDN), established in 2010, with the aim of further extending the health and other development issues to household and individual levels (14–16). The WDN has a broad mission to empower women and improve their status by bringing information and services to their fellow women. To this effect, the WDNs have been organized across the country and engaged in several development affairs such as health, economics, education, and politics to mention some (17).

Studies have revealed that the WDN has contributed to the betterment of women in certain aspects (14, 16), but not adequately sorted and revealed as to what extent and depth these improvements and how sustainable they would be under the dynamic sociopolitical and economic circumstances (17). In a similar token, the functionality of the WDN is not free from challenges and drawbacks. Studies indicated that grassroots development agents such as the WDN need proper handling for their effective function (18–23). Members of the WDN are women themselves with numerous domestic roles and responsibilities. Their volunteer WDN services further burden their roles (14). Consequently, exploring the pattern of functionality and factors contributing to its functionality to strengthen the loose links and promote the positive attributes to the wider development aspects becomes imperative.

## Materials and methods

### Study area

The study was conducted in Southern Nations, Nationalities and People's Region (SNNPR). The region was known for its ethnic, cultural, linguistic, and geographical diversities. Based on the population projection of 2007, the regional population was estimated to be 18 million (24, 25). The region is one of the most densely populated regions of the country. After data collection, the region is divided into three regions: South Ethiopia, Central Ethiopia, and Southwest Ethiopia. The WDN is composed of six one-to-five networks of neighborhood women, each representing a household. The one-to-five network is called the Women’s Development Army (WDA). In the WDA, one represents the leader, and five represent the members led by the leader. The WDN, therefore, comprises, on average, 30 households. The WDN leaders were trained on the health extension program packages by the health extension workers for about 60 days, which was supported by the catchment health center.

### Study design and period

An exploratory qualitative design was employed for the study in 2021 as it provides the researcher with a means of understanding a phenomenon by observing or interacting with the participants of the study (26, 27). Therefore, qualitative researchers are interested in exploring and/or explaining phenomena as they occur in the natural setting. In connection with this, the exploratory qualitative design is appropriate to explore and describe aspects of the WDNs.

Scientific evidence generation entails various approaches over time (28). The early empirical study-dominated paradigm was mainly focused on the qualitative methodology and related data collection methods. As human query develops over time, the human world outlook expands and grows. Consequently, the constructivist paradigm existed due to human nature, which is beyond equating everything to statistics/numbers. Human desire and nature are beyond figures that can be investigated qualitatively considering the history, culture, livelihood, prospective, and other complicated human variables. The constructive paradigm in its turn uses a qualitative study design and its respective data collection methods such as focus group discussions (FGDs), key informant interviews (KII), and individual in-depth interviews (III) (29). Therefore, this study employed an exploratory qualitative design. This approach enables us to conduct qualitative data at the same time and help analyze it concurrently. The superior advantage of this approach is that it helps to comprehend the detailed aspects of the study questions and its objectives.

### Study population and sampling

The source populations for this study included women in the region organized under the WDNs, leaders at various levels from the kebele to the region inclusive of district/woreda, special woreda, and zone. Woreda, sometimes called district, is an administrative structure of about 100,000 population while kebele is the smallest administrative structure with an approximate population of 5,000. Since the SNNPR was administratively classified into zone, woreda/special woreda, and kebele, the sampling process considered the existing hierarchies. There were 16 zones, 7 special woredas, and 190 woredas. The WDN in the region were graded with “A,” “B,” or “C” based on the WDN's functionalities and performance: “A” being the best performance and “C” being the least. The sample zones/special woredas were selected purposively as presented Table 1.

**Table 1:** Sampling sketch for study zones and special woredas.

Cluster	Zones/special woreda	Remark
South cluster	Wolaita	Two woredas from two zones/special woredas were considered
	Gammo	
	Goffa	
	Konso	
	Allie	
	Derashe	
	South Omo	
	Basketo	
Central cluster	Guragie	Two woredas from two zones/special woredas were considered
	Hadya	
	Kembata-Tambaro	
	Silitie	
	Halaba	
	Yem	
West cluster	Kaffa	Two woredas from two zones/special woredas were considered
	Dawuro	
	Benchi Sheko	
	West Omo	
	Sheka	
	Konta	
East cluster	Gedeo	One woreda was selected
	Burji	
	Amaro	

At the kebele level, community members (FGDs), WDN leaders, women’s federation representatives, kebele administrators, and health extension workers (HEW) were interviewed. At the woreda level, experts from the health office, administration office, and Women, Children and Youth Affairs (WCYA) office were interviewed. At the zonal and regional levels, the health sector, WCYA, chief administrative office, and counsel were included (Table 2).

**Table 2:** List of data sources.

Administrative hierarchy	Data source	Method of data collection	Total size
Kebele	Women in the community	FGD	14
	WDN leaders	III	14
	Women’s federation representative	KII	14
	Kebele administrator	KII	14
	HEW	KII	14
Woreda	Health office	KII	7
	WCYA office	KII	7
	Administrator office	KII	7
Zone	Health department	KII	7
	WCYA department	KII	7
	Chief admin office	KII	7
Region	Health bureau	KII	1
	WCYA bureau	KII	1
	President's office	KII	1
	Regional council	KII	1

### Methods of data collection

The data sources for the FGD were women organized under the WDN in their respective groups. A total of 14 FGDs were carried out across the entire region based on the sketch shown in Tables 1 and 2. In each FGD, at least 8 and at most 12 women were involved. A total of seven individual in-depth interviews were conducted: one in each kebele. The source of the KI information was informants in enrolled organizations ranging from the kebele to the region due to their acquaintance with the WDN.

### Methods of data analysis

Audio-taped materials were transcribed verbatim. Transcriptions and notes were read several times to acquaint with data and find repeating terms and concepts that convey similar meanings. Such words and concepts were identified and coded initially as open code. The coding process allowed for the further interpretation of large segments of text and portions of information. Assessing how these meaning units were linked; led to the identification of themes. The steps were conducted repeatedly, thus resulting in the creation of a very large number of codes and themes. The coding and thematizing followed by meaning-making interpretation of the findings. The process was enriched by literature consultation to find qualitative data. Finally, the findings were narrated according to the study objectives.

### Ensuring trustworthiness

Data quality was ensured by taking all necessary measures help to maintain its quality. The trustworthiness of the data was ensured through proper and meticulous handling of all the steps from the proposal development to the final write-up. More emphasis was given to properly moderate the discussion and generate rich information from the interviews. The development of sound study guide questions and validation of the questions among various professional groups or principal investigators guaranteed the quality. Vigilance in transcribing the audio-taped materials verbatim and cross-checked by principal investigators. To ensure the data collection process toward quality, all the audio materials were checked for functionality, and the convenience of rooms for the FGD, KII, and individual in-depth interviews was given adequate attention.

### Ethical considerations

Ethical clearance for this study was obtained from the Institutional Review Board (IRB) of SNNPR Public Health Institute (Ref እን 1–5/9002). A support letter from the health bureau (Ref. የወ6–19/1859) was taken to respective study zones, woredas, and kebeles. Written consent was obtained from each study participant to confirm that participation in the study was purely voluntary. Detailed information was provided for the participants to ensure their rights of participation. However, the participants were encouraged that their participation in the study was crucial for the general improvement of the WDNs thereby empowering women through overall engagements in development activities. All the data were kept confidential and anonymous.

## Results

### Contributions of WDN to development

The WDN was designed to improve the multidimensional women’s development needs. The structure functions in the entire nation including the SNNPR. All participants agreed that WDN has contributed multidimensional development inputs. It has contributed to improvements in household welfare resulting from increased ability to afford food, clothing, health, and education. As WDN is closely trained and supervised by female health extension workers, the contribution of WDN to health has been bold. To health affairs, WDN has contributed to general awareness creation, maternal and child health utilization, and environmental sanitation. More specifically, WDN has been playing a crucial role in identifying and referring sick children, identifying defaulters from immunization and maternal health services (such as antenatal care, institutional delivery, postnatal care, and family planning), constructing latrines, and so on. Related to this, WDN has been playing a key role in planting vegetables in the garden.

Mainly, WDN focuses on mothers especially on promoting health under the guidance of health extension workers. Focusing on women is because of their tight linkage with their families. Generally, they have been working on 16 packages but currently it was raised to 18 packages especially applied in rural area. In addition, WDN works to help mothers to improve knowledge, skill and attitude of individual women in their respective organization on different health issues. This group mainly shares messages regarding the antenatal and postnatal follow-up, family planning services, vaccination, nutritional assessments follow-up and other packages. (KI-3)

WDN contribute to prevention of malaria, maternal health and child health issues, malnutrition prevention and treatment like outpatient therapeutic programs and in facilitating vaccination uptakes. WDN also mobilizes the community for immunization and facilitates family planning uptakes. (KI-5)

### Patterns and contributing factors of WDN functionality

The study has explored the patterns of WDN functionality and factors affecting this functionality in the region. The study further explored what should be done to strengthen the WDN. As it is substantiated by the word of the study participants:

I have mentioned important points that made WDN strong. All stockholders and organizations from the community level to the regional level follow the functions of WDN. The structure received remarkable attention of the government and the community at its inception and early time of functions. (KI-1)

The other point is every sector that has a concern or stake in the WDN should be identified with its role and responsibilities. I personally believe WDN should be implemented if women need to be active in the economy, socially and politically. Hence, the renewal action should steer all stakeholders to discharge their role and responsibilities. (KI-2)

Actions shall be practical and effective by all stakeholders working towards the desired levels of WDN performance. The other thing is whatever the answer, the question of 1 to 5 teams and WDN should get feedback. If so, it can be active more than before. (KI-3)

WDN in its due course experienced staggering steps and, in the middle, attained firm status with good acceptance by the community and serving them in a desirable manner. Over time, the strength and acceptance were drained. Consequently, study participants reflected on their observations and recommendations for what to be done to strengthen and revitalize the WDN. It is obvious that any development endeavor needs proper handling and nurturing for lasting functionality. WDN is not an exception. The direct words of the participants supported the assertion in the following way:

The program should be reviewed, and some drawbacks should be corrected and implemented as full package to benefit all the women. WDN and the 1 to 5 organizational members should be encouraged to make effective participation in their membership to achieve their planned activities. (KI-22)

The reason for weakness is the absence of follow-up. If there is no follow-up, how we can be active? Mind you that we are serving with limited capacity in a volunteer manner in the growing demands of the fellow women we serve. As the result, we need to receive refresher training, encouragement and moral support. However, such undertakings are not in place as required. (FGD SO-1)

Revitalizing WDN's strength as its inception period strength has become a timely need. As it was explicated by the study participants, the establishment of WDN was done with the boiling motives of several stakeholders across the nation and the regions. The steps were similar in the SNNPR. All the strong points should come back to their proper positions and the drawbacks systematically be corrected.

There should be desirable level of commitment and coordination between stakeholders for planning, monitoring, and evaluating the achievements. There should be a stage at woreda level for experience sharing, learning from each other and resolve dependency on the upper structure so that they can perform the activities by themselves. (KI-23)

To sustain the functions of WDN, continuous refreshment training with increasing depth and complexity is to be given in a demand-based and flexible manner. Training and the performance after the training should be monitored, followed, and evaluated. Moreover, supportive supervision by the health extension workers, kebele administrator, and woreda-level stakeholders should be in a regular fashion. Development army members and leaders are women themselves with numerous domestic and family responsibilities. This volunteer task (WDN's role) they provide in the community tightens their situations. It means they have an increased workload in their house and the community. Unless properly rewarded, it is imminent that WDN will remain weak. Therefore, all stakeholders closely functioning with WDN, must contribute to their empowerment. The rewards should be composed of financial and non-financial such as creating a career path, morale boosting, and encouragement. This is substantiated by the direct statements from the study participants as follows:

Very small incentive package can make a huge difference. Providing trainings and meetings with refreshment, providing stationeries for reporting purpose, and encouraging best performing WDN’s is quite enough intensive. Training for leaders of WDNs who have completed grade 10 can substitute health extension workers and dramatically shift positively the implementation of the health extension packages. Calling and coordinating resources from stakeholders, including non-governmental organizations, can positively improve the performance WDNs. (KI-27).

The government usually sends agendas to the WDNs without any logistics and financial support. This needs to be modified and financial support is required from the government side. In addition to this, we don't see the government recognizing the program. This curtails WDNs progress on their tasks and accomplish in desired time. (KI-33)

A one- to-five administrations must be advocated well by women. Most of the time when women are asked to come for meetings, they would let their homework undone. They are doing this job without payment. So, it would be better if the 1 to 5 leaders were given some incentives so that they will be encouraged and motivated. Our main target is educating the community and showing the benefits. We are putting the women on the right track. So, to get a better result something must be done. For example, those women who passed the COC exam were believed to replace the health extension workers. So, in the future, if something is done for these mothers, I expect they will participate more. (KI-50)

Alongside the efforts to further consolidate the WDN, maintenance of the loose links includes bringing stakeholders such as men (husbands, educated women, civil servants) who see the WDN skeptically on board and establish shared understandings and win their confidence. Involving these groups in the community would help the WDN to function comfortably and confidence.

Currently we have 27 WDNs but as per the number of the population, we have the potential of reaching 30 WDNs. Moreover, currently only 14 are active while the remaining 13 (almost half) are passive. The implication is that organizing new WDNs should continue parallel to activating passive ones. Woreda health offices should organize training for WDN leaders, with refreshments and reimbursement for their time spent in trainings. Awareness creation trainings for men (husband) can increase the involvement of women (wives) in the activities of WDNs. (KI-28)

The weakness of WDN symbolizes the weakness in the health extension program. More so is evidence of the lack of a mainstream system. Therefore, strengthening WDN first begins by strengthening the HEP and then by mainstreaming the WDN into the related sectors. The lack of clear responsibilities and accountability among the involved stakeholders related to WDN has been creating confusion not only for the stakeholders but also for the WDN itself. The multiple command systems and the lack of proper collaboration and coordination among these sectors are the leading causes of the observed confusion and disagreements.

After the government reform, the WDN networks started to weaken. The new road map is ready to be launched by Ministry of Health. Motivating HEWS and WDN leaders is fundamental for the sustainability of the programs. Supporting HEWs' home to home visit by transportation, arranging a transfer for HEW and changing the size of 1 to 5 networks can improve the structure. (KI-37)

Therefore, it needs to have well-coordinated, clearly apportioned roles, responsibilities, and accountability for all the involved stakeholders in relation to the WDN management. It also necessitates having a responsible wing at the kebele level directly supporting the WDN by representing the women, children, and youth affairs sector. Furthermore, the WDN structure should be revisited with a clear career path based on their experiences and educational preparedness. The monitoring and evaluation of WDN performance should be accounted for to differentiate the best performing from the weakest and equivalent rewards have to be instituted for the best performing.

To enrich the experiences and learn from others, the WDN management system has to think about creating experience-sharing platforms across the kebeles and woredas in the nearby vicinities. Political involvement should be refrained to the minimal as the structure is largely for the development endeavor and serves all in a secular manner. If this is maintained, acceptability by the broader community will be improved and far-reaching. Otherwise, the existing suspicion from segments of the community and that of the opponent political groups will continue.

Perhaps, based on this study and other further investigations, new structure should be done. The other things are training to create and increase awareness and follow-up the accomplishment and monitoring and evaluation work (including feedback) are mandatory remedial actions. (One of the FGDs discussants)

What makes WDN weak is a political issue, not COVID-19. The solution also should come from politics. Politicians should not use this 1 to 5 and WDN for their political game, only for development and creating consciousness of women. The other thing is the mandate of organizing WDN and women's 1 to 5 should be clear. Once, one team from the region come here and told the mandate of organizing women is the Women, Children and Youth (WCY) office, the other wing says it is not their mandated to organize. This needs clarification. If we do not have a mandate, why should we be blamed for the failure of WDN and women’s 1 to 5? (KI-25)

## Discussion

The success and sustainability of any program are contingent on how the program is managed. Unwavering follow-up, monitoring evaluation, and feedback are crucial for the success of a given program (17, 18). Similarly, WDN was designed with the broad aim of improving the multidimensional women’s development needs in the entire nation including the SNNPR.

WDN in its due course experienced triple phases of which the staggering steps at its early stage, better functioning in the middle, and similar staggering recently. Over time, the strength and acceptance were drained. Consequently, study participants reflected on their observations and recommendations for what to be done to strengthen and revitalize the WDN. It is obvious that any development endeavor needs proper handling and nurturing to last longer and yield productively. WDN is not an exception (17, 18).

The inception period strength of WDN should be revitalized. As it was explicated by the study participants, the establishment of WDN was done with the boiling motives of several stakeholders across the nation and the regions. The steps were similar in the SNNPR. All the strong dimensions should come back to their proper positions and the drawbacks systematically be corrected.

The sustainability of WDN functionality could depend on the continuous refreshment training with increasing depth and complexity. Such provision should be demand based and in flexible manners. Moreover, the training provided and the subsequent performance should also be monitored, followed, and evaluated. This finding is commensurate with the study finding by Damtew et al. (2018) (15), which states that higher WDN strategic implementation was associated with better healthcare behavior and practice, that the WDN strategic support received from the HEWs also improves the performance and motivation of the WDN.

WDN members and leaders are women themselves with numerous domestic and family responsibilities other than the WDN roles and responsibilities (13, 14). The volunteer nature of the tasks in the community connected to their low livelihood status created a tight socioeconomic situation. It means that they have an increased workload both in their house and in the community.

Unless rewarding mechanisms are in place, it is imminent that WDN will remain weak. Therefore, be it the kebele administrator, the woreda-level sectors in one way or another closely function with the WDN and must contribute to their empowerment. The rewards may be composed of financial and non-financial such as creating a career path, morale boosting, and encouragement. In connection with this finding, Closser et al., (2020) (13) indicated that unless careful attention is paid to address the socioeconomic and psychological needs of such groups, those who are already living in a precarious situation would further be devastated.

Moreover, the efforts to further consolidate the WDN's functionality should further consider bringing on board stakeholders such as husbands, educated women, and civil servants who see the WDN skeptically by establishing shared understandings. Involving these groups of community would help the WDN to function comfortably and with confidence. Educated women tend to take leadership positions, specifically within the community (30). In some settings, even they do not want to participate in community development projects (31). However, due to the free volunteer nature of WDN, there could be a challenge to engage educated women in the WDN leadership roles. This, thus, requires sharing and convincing of societal development goals.

WDN’s weakness is closely connected to the weakness in the health extension program, which is revealed by the absence of a strong system. Therefore, strengthening WDN should begin first by strengthening the HEP and then by mainstreaming the WDN into the related sectors. The finding of this study echoes the study finding by Assefa et al., (2019) (13) that states that the challenges related to the working conditions, capacity of health posts, elements of motivation, and rewards affect the productivity and efficiency of the health extension workers. The lack of clear responsibilities and accountability among the involved stakeholders related to WDN has been creating confusion not only for the stakeholders but also for the WDN itself. The multiple command systems and the lack of proper collaboration and coordination among these sectors are the leading causes of the observed confusion and disagreements. This finding is similar to that of studies by Fetene et al. (2020) and Genovese et al. (2017) that state that accountability when functioning properly helps reduce abuse, assure compliance with procedures and standards, and improve organizational goals (19, 20). The absence thereof creates an untoward effect and leads to confusion among the acting stakeholders. As such, accountability in primary healthcare in Ethiopia needs improvement.

The finding indicated clear gaps in coordination and the absence of clearly apportioned roles, responsibilities, and accountability for all the involved stakeholders in relation to the WDN management. The lack of a responsible wing at the kebele level directly supporting the WDN by representing the women, children, and youth affairs sector was observed. Furthermore, the WDN lacks a clear career path based on their experiences and educational preparedness. The monitoring and evaluation of WDN performance are ill-instituted and unable to differentiate the best performing from the weakest and equivalent rewards must be instituted for the best performing. This study finding is in line with the study conducted by Ashebir et al. (2021) and WHO (2017) in Tigray, and the entire nation region has indicated that lack of proper supervision, follow-up, and rewards to the women’s development team has been the bottleneck for the desirable contribution of the team despite the clear rationale for their establishment (22, 23).

Experiences are less communicated or shared with one another to enrich the WDN management system. As this study has indicated, there is no such experience-sharing platform across the kebeles and woredas in the nearby vicinities. On the other way, the study revealed huge political involvement, thus overburdening WDN with their mission whereas the structure is largely for the development endeavor and has to serve all in a secular manner. With the huge political interference in the WDN, the acceptability by the broader community would be challenged. Furthermore, the existing suspicion from segments of the community from opposing political groups will continue. In a similar manner, Maes et al. (2018) indicated that the pervasive political engagement in the functionality of WDN and its management affected their empowerment in contrary as it was just a rhetoric (16).

### Limitations of the study

The study has employed an exploratory qualitative design. This study design bears several advantages yet is not free from limitations. Consequently, this study also faces similar limitations like lack of quantitative data rigors and inability to set generalization of the findings other than the study areas. The study might not benefit part of the regions not included in the study and desire to conduct subsequent studies based on the recommendations. In addition, even though all participant women were neighbors from similar settings, the knowledge and attitude of the women involved could be linked to their educational backgrounds. However, the enrollment process did not consider it in the criteria for selection and that could affect others’ participation in discussion. Moreover, although poor men’s involvement in the network has been raised as a factor for WDN weakness, the role of men so far has not been assessed.

## Conclusion

The study explored that WDN was established with heightened assumptions and enthusiasms to further strengthen women’s involvement in the multidimensional development arena. It has received the attention of several sectors, partners, and stakeholders. The health sector is the first one followed by the women and children affairs sector and other government organizations and partners.

WDN has contributed significantly to mobilizing their fellow women and helped them to engage in social, economic, political, and other related dimensions. WDN has contributed to the improvements in household welfare resulting from an increased ability to afford food, clothing, health, and education-related costs. Clearly, WDN has enabled some female participants to enter informal saving associations and access credit enabling some of them to pursue economic investments.

However, the enthusiasm for WDN becomes blurred over time due to several factors. The initial strong support, refreshment training, and follow-up gradually weakened. Lack of proper reward and compensation for the time and energy women invest in the WDN function eventually overburdened compete with their daily domestic affairs. Some groups of society see the WDN function skeptically and as a waste of time. They underestimate WDN's contributions. On the other hand, WDN members and leaders are overstretched due to the assignments they are given other than the initial purposes for which they are mandated. Consequently, WDN functions deteriorate eventually to the extent that they would be stalled in a short time unless corrective measures could be taken.

Considering such vital inputs from community participation in resource-limited settings, efforts are required to maintain the strength and enthusiasm of WDN to its original state and ensure the multidimensional benefits for their fellow women from the program:

• All the stockholders and organizations should act in a harmonized way discharging their responsibility effectively towards WDN.
• Strengthen the supportive supervision, follow-up, timely feedback, and need-based refresher training.
• Proper mechanisms should be in place to motivate WDN members by identifying their economic, training, and other relevant needs directly aligned to their mission, thereby reducing overburdening of assignments.
• Establish an objective evaluation system, weigh their performance, and create a clear career path to sustain the program.
• Strengthen the health extension program, which is the backbone for the existence and strength of the WDN.

## Data Availability

The raw data supporting the conclusions of this article will be made available by the authors, without undue reservation.
